# Induction of *PR-10* genes and metabolites in strawberry plants in response to *Verticillium dahliae* infection

**DOI:** 10.1186/s12870-019-1718-x

**Published:** 2019-04-05

**Authors:** Fatma Besbes, Ruth Habegger, Wilfried Schwab

**Affiliations:** Biotechnology of Natural Products, Technische Universität München, Liesel-Beckmann-Str. 1, 85354 Freising, Germany

**Keywords:** *Fragaria × ananassa*, Strawberry, *Verticillium dahliae*, PR-10, Gene expression, qPCR

## Abstract

**Background:**

The soil-borne vascular pathogen *Verticillium dahliae* causes severe wilt symptoms in a wide range of plants including strawberry (*Fragaria × ananassa).* To enhance our understanding of the effects of *V. dahliae* on the growth and development of *F. × ananassa,* the expression patterns of 21 *PR-10* genes were investigated by qPCR analysis and metabolite changes were determined by LC-MS in in vitro *F. × ananassa* plants upon pathogen infection.

**Results:**

The expression patterns of the 21 isoforms showed a wide range of responses. Four *PR-10* genes were highly induced in leaves upon pathogen infection while eight members were significantly up-regulated in roots. A simultaneously induced expression in leaves and roots was detected for five *PR-10* genes. Interestingly, two isoforms were expressed upon infection in all three tissues (leaves, roots and stems) while no induction was detected for two other members. Accumulation of antifungal catechin and epicatechin was detected upon pathogen infection in roots and stems at late stages, while caffeic acid and citric acid were observed only in infected roots. Production of abscisic acid, salicylic acid, jasmonic acid (JA), gibberellic acid and indole acetic acid (IAA) was induced in infected leaves and stems at early stages. IAA and JA were the sole hormones to be ascertained in infected roots at late stages.

**Conclusions:**

The induction of several *PR-10* genes upon infection of strawberry plants with *V. dahliae* suggest a role of *PR-10* genes in the defense response against this pathogen. Production of phytohormones in the early stages of infection and antifungal metabolites in late stages suppose that they are implicated in this response. The results may possibly improve the control measures of the pathogen.

**Electronic supplementary material:**

The online version of this article (10.1186/s12870-019-1718-x) contains supplementary material, which is available to authorized users.

## Background

Phytopathogenic fungi of the genus *Verticillium* cause billions of annual damage to agricultural crops worldwide [1]. The most studied species among the ten species currently grouped in the genus *Verticillium* sensu stricto is *Verticillium dahliae* because it is responsible for huge economic losses in crops in many regions of the world [2–4]. A plant species, which is highly susceptible to severe disease caused by the soil-borne pathogenic fungus *V. dahliae* is the cultivated strawberry (*Fragaria × ananassa*) plant. *V. dahliae* was first described from Dahlia sp. cv. Geiselher and has the largest impact as a pathogen as the probability of surviving infection by the *Verticillium* wilt fungus to produce a crop is greatly reduced and the control of *Verticillium* wilt is difficult and costly [5–7]. In the absence of a suitable plant host, *V. dahliae* remain dormant in the soil for years by means of small melanized resting structures (microsclerotia) and germinate only in the proximity of a suitable host [8].

The life cycle of the phytopathogenic fungi comprises three vegetative phases: dormant, parasitic and saprophytic. In the dormant phase, the nitrogen- and carbon-rich root exudates [9] stimulate germination of microsclerotia, which are long lasting multicellular structures. These structures enable *Verticillium* spp. to infect susceptible plants and enter their parasitic stage. Infection occurs either at the sites of lateral root formation or at the root tip [10]. After infiltration of the vascular cylinder the phytopathogenic fungi begins to form conidia, which travel in the xylem system [11, 12]. The plant becomes increasingly colonized, begins to wilt and develops typical symptoms of necrosis. The fungus enters a limited saprophytic growth phase in which microsclerotia are formed. When released into the soil the microsclerotia can survive for 10–15 years [8]. Monocyclic pathogens such as *V. dahliae* go through only one round of disease and inoculum production per growing season and do not need to repeat the cycle in subsequent years [1, 13].

Plants can respond to both biotic and abiotic stresses. Pathogenesis-related (PR) proteins, including family 10 proteins (PR-10), are induced under biotic and abiotic stress and are therefore involved in plant defense [14]. The PR-proteins are expressed in many plant species e.g. after fungal infection during induction of resistance. In cotton, early stage expression of PR-10 combined with phytoalexin production contributes to *Verticillium* wilt resistance [15]. PR-10 proteins are encoded by multigene families and are constitutively expressed in different organs. Numerous functions are assigned to this protein family [16].

Despite several studies and investigations, the specific role of individual PR-10 members remains unclear. This work aimed to investigate the molecular interplay between in vitro strawberry plants and the *V. dahliae* pathogen in order to get molecular evidence about strawberry defense response, to identify induced *PR-10* genes upon pathogen infection and to achieve insight into the hormonal response in strawberry. The purpose of the current study was to contribute to the understanding of the function of the *PR-10* isoforms.

## Results

### Visual evaluation of disease severity and fungal propagation

**Fig. 1:**
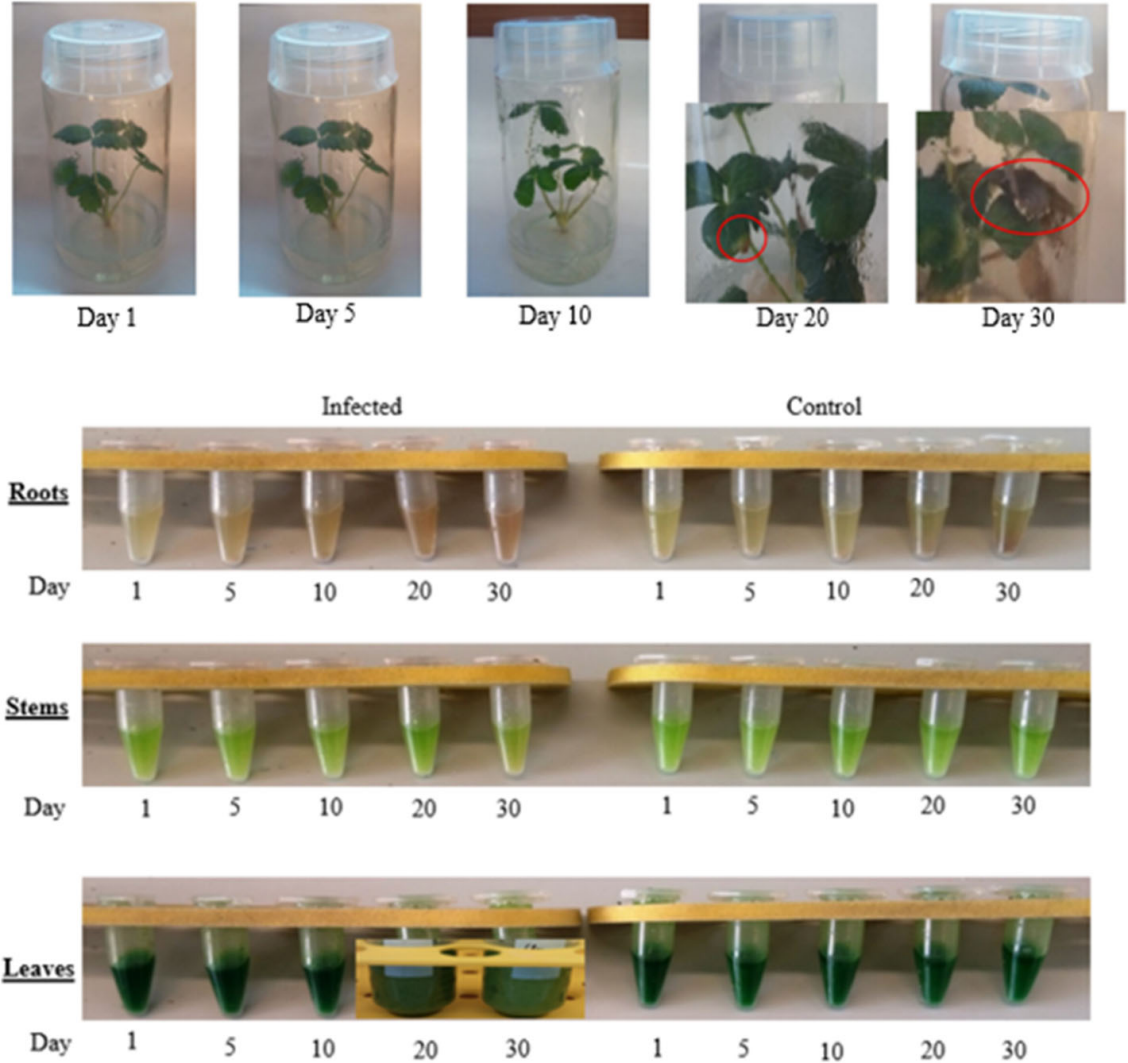
Development of disease symptoms in in vitro plants infected with *V. dahliae* at different time points (1, 5, 10, 20, and 30 days) and comparison of methanol extracts of lyophilized tissues from control and infected organs at different time points post *V. dahliae* infection (1, 5, 10, 20 and 30 days)

*F. × ananassa* in vitro plants were artificially inoculated with the isolate E650 of *V. dahliae* (Additional file 1). At all sampling time points, plants were visually assessed for the appearance of foliar symptoms using a 0–5 scale according to [17]. The scale is based on the percentage of diseased leaf area. Disease symptoms developed gradually and became visible 20 days post *Verticillium* inoculation. Scale 2 (10–25% of symptomatic leaf area) was attributed to plants at 20 dpi (days post infection) and scale 5 (75–100% of symptomatic leaf area) for plants at 30 dpi. For control and infected plants during the first 20 days, no symptoms were observed and therefore scale 0 was assigned (Fig. 1). Samples were also evaluated based on color modification after methanol extraction of lyophilized tissues (Fig. 1). Comparison of *Verticillium* infected roots and the control demonstrated that the color was yellowish at the first day and became progressively light brown at 30 dpi for the infected roots and yellowish brown for the control. Similarly, a color change was visible between 1 dpi (fluorescent green) and 30 dpi (fluorescent yellowish green) in infected stems but not for the control tissue whereas in infected leaves, the unique color change occurred at 20 dpi (dark green) and 30 dpi (light green) (Fig. 1). No color change was detected for the control leaves.

### Validation of primer design

BLAST analysis identified 21 *PR-10* genes in the genome sequence of *F. vesca* ([18]; Additional file 2). Because of the high sequence similarity between the 21 *PR-10* isoforms (nearly 70% of pairwise nucleotide identity), specific primers for quantitative polymerse chain reaction (qPCR) were designed using the 5′ UTR and 3′ UTR regions (Additional file 3). The qPCR analysis of *PR-10.01-03* was performed using the specific primer pairs as described [19]. For all other *PR-10* genes specific primers were designed using the 5′ UTR, except for *PR-10.09*, *PR-10.14*, *PR-10-17* and *PR-10.20*. For efficient amplification in qPCR, primers used in this study produced amplicons of sizes less than 200 bp. Except for the reverse primer of *PR-10.05,* which had a length of 19 bp, all the remaining *PR-10* primers showed sizes between 20 and 27 bp. The GC content varied from 32 to 65%. In addition, apart from the primer of *PR-10.16*, which had a melting temperature (Tm) of 56 °C, Tm for the primers of the remaining *PR-10* isoforms were between 59 °C and 64 °C. The gene specificity of the designed primers was confirmed by Basic Local Alignment Search Tool (BLAST) analysis of the primer sequences (forward and reverse) of each of the 21 *PR-10* isoforms. Each primer perfectly matched only the sequence of the target gene. Even though the primers were designed on the *PR-10* sequences from *F. vesca*, they also produced respective amplicons from *F. × ananassa.* The melting curves recorded after the transcript analyses showed single distinct peaks indicating high specificity for all *PR-10* isoforms.

### Quantitative real-time PCR analysis of 21 *PR-10* genes in response to *Verticillium* infection

**Fig. 2:**
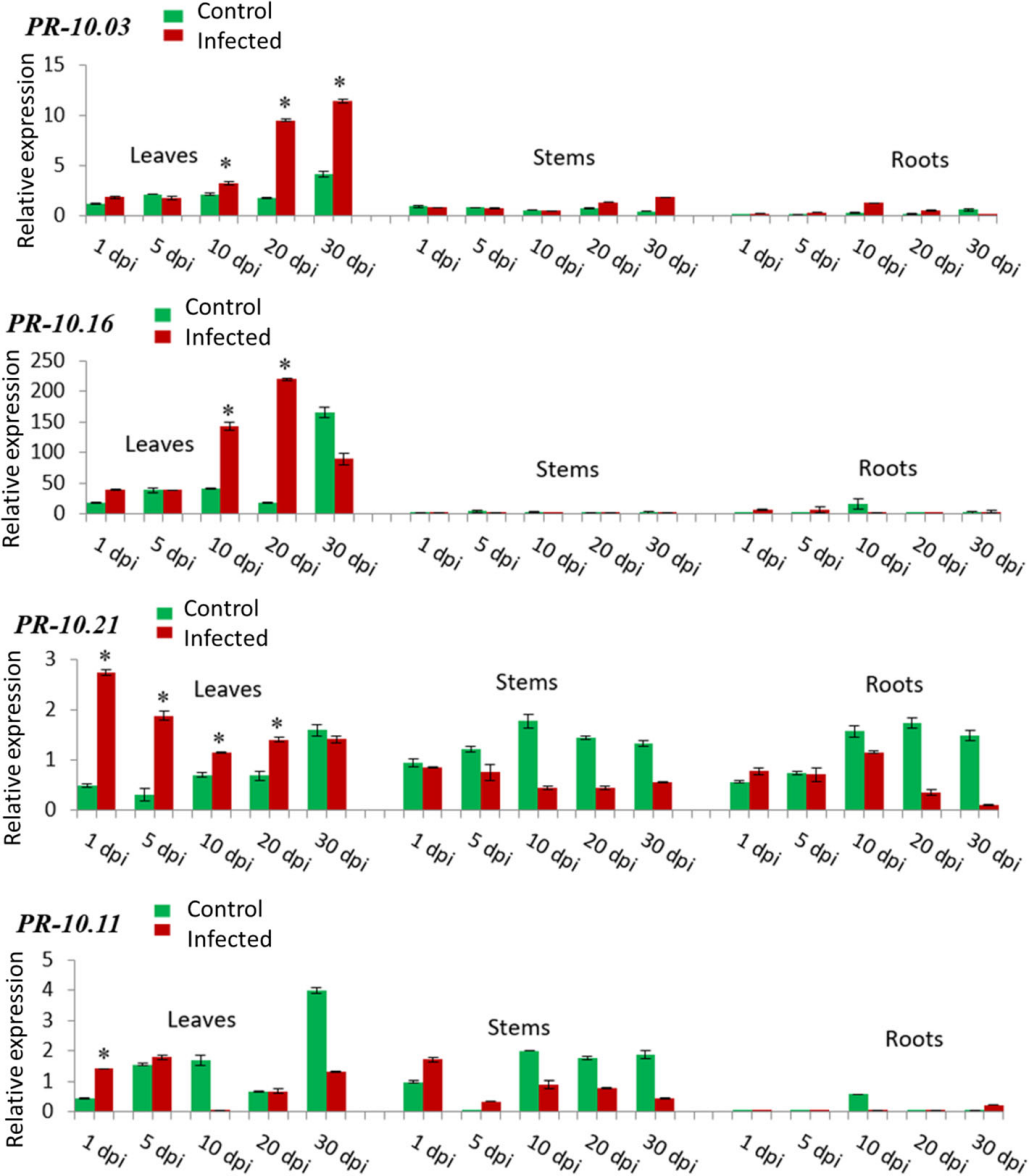
Relative transcript levels determined by qPCR of PR-10.03, PR-10.11, PR-10.16 and PR-10.21, which are highly induced in leaves. Strawberry plants were infected with *V. dahliae* and the relative transcript levels were normalized to the control stem at 1 dpi (set to one). The expression levels in the tissues were measured at 1, 5, 10, 20, and 30 dpi (days post infection). Green bars represent the control while red bars represent infected tissues. The mean values (± SD) were obtained from five biological replicates and are shown as relative changes. Asterisks represent significant induction of a gene after statistical comparison with the transcript abundance of the control sample using the Tukey test. FaRIB413 was used as a housekeeping gene for normalization

To examine the effect of pathogen infection on the expression of *PR-10* genes, in vitro cultivated strawberry plants were infected with *V. dahliae*. Samples were taken at defined intervals post infection (dpi). Non-infected plants served as control. Values were calculated as fold change of each sample relative to a reference gene (*FaRIB413*). The fold changes of each transcript (relative values) upon *Verticillium* infection were calculated and represented for each *Fra a 1* gene. The expression pattern of 21 *PR-10* isoforms in different tissues (leaves, stems and roots) of the strawberry plants, after infection with *V. dahliae* showed a wide range of response. *PR-10.03* (11.4 fold relative expression at 30 dpi) and *PR-10.16* (219.9 fold rel. Exp. at 20 dpi) were significantly upregulated in leaves after pathogen infection (Fig. 2). This induction increased through the time and reached a maximum after 20 to 30 days of infection. For *PR-10.21* (2.7 fold rel. Exp. at 1 dpi), the highest induction level was detected in leaves already after 1 day of infection followed by a steady decrease until the 10th day. For *PR-10.11* (1.4 fold rel. Exp. at 1 dpi), the induction of expression was also significant in leaves at day 1 post inoculation (Fig. 2).

**Fig. 3:**
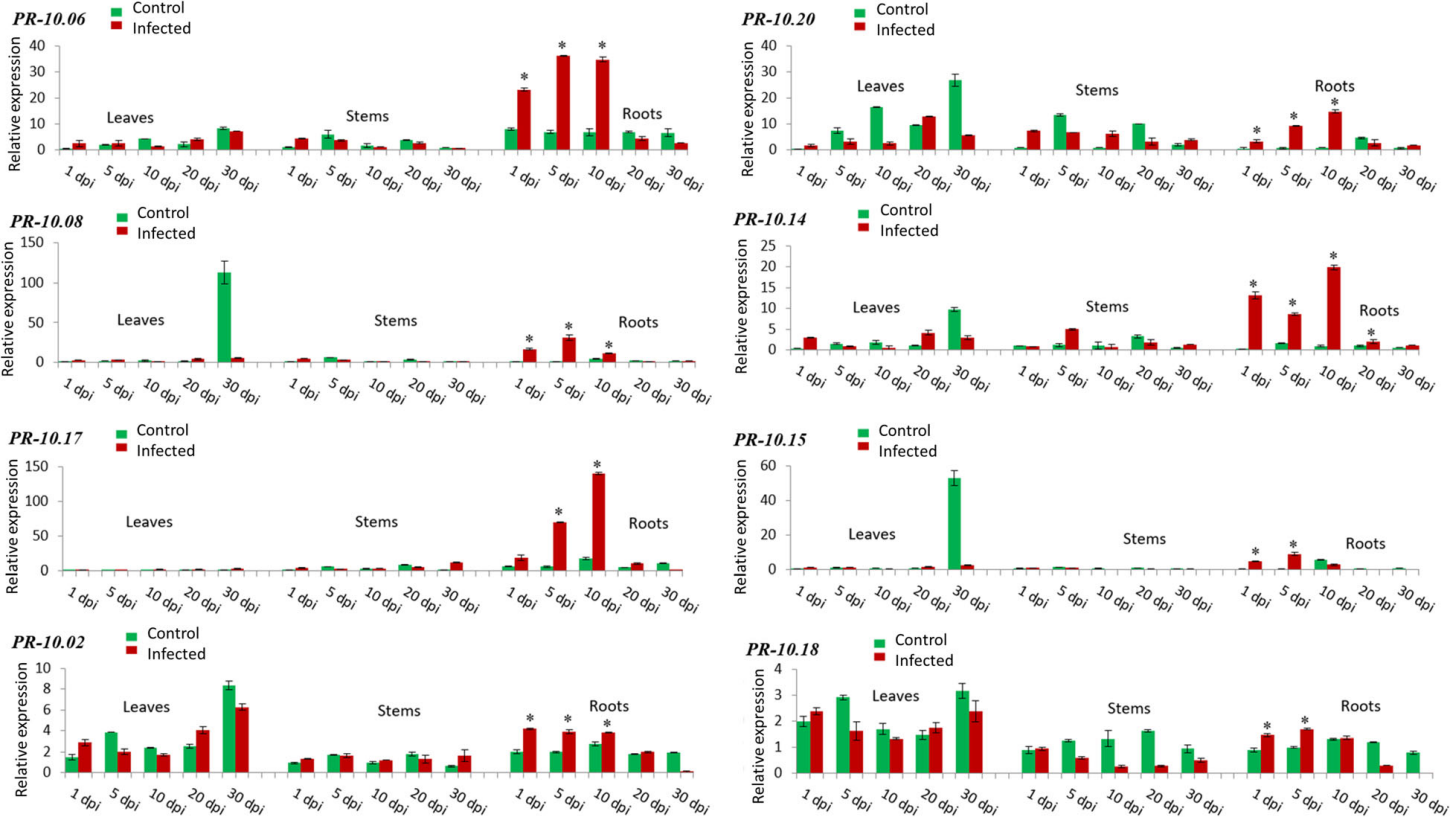
Relative transcript levels determined by qPCR of *PR-10.06*, *PR-10.08*, *PR-10.17, PR-10.02*, *PR-10.20*, *PR-10.14*, *PR-10.15* and *PR-10.18, which* are highly induced in roots. Strawberry plants were infected with *V. dahliae* and the relative transcript level was normalized to the control stem at 1 dpi (set to one). The expression levels in the tissues were measured at 1, 5, 10, 20, and 30 dpi (days post infection). Green bars represent the control while red bars represent infected tissues. The mean values (± SD) were obtained from five biological replicates and are shown as relative changes. Asterisks represent significant induction of a gene after statistical comparison with the transcript abundance of the control sample using the Tukey test. *FaRIB413* was used as a housekeeping gene for normalization

*PR-10.06*, *PR-10.08* and *PR-10.17* were highly expressed in roots after pathogen infection (Fig. 3). There was an up to 36.1 fold relative transcript level for *PR-10.06*, 31.3 fold rel. Exp. for *PR-10.08,* and 140 fold rel. Exp. for *PR-10.17* at 10 dpi. The induction was more relevant at day 5 for *PR-10.06* and *PR-10.08* and at day 10 for *PR-10.17.* Interestingly, a quick and prominent upregulation was also observed in roots for *PR-10.02* (4.2 fold rel. Exp.) at 1 dpi which declined slightly over time. Similarly, *PR-10.20* (14.8 fold rel. Exp. at 10 dpi) and *PR-10.14* (19.8 fold rel. Exp. at 10 dpi) were highly induced in roots but the maximum peak occurred at day 10. Moreover, the expression patterns of *PR-10.15* (9.0 fold rel. Exp. at 5 dpi) and *PR-10.18* (1.7 fold rel. Exp. at 5 dpi) were similar in roots and increased from day 1 to day 5 followed by a diminution until day 30 (Fig. 3).

**Fig. 4:**
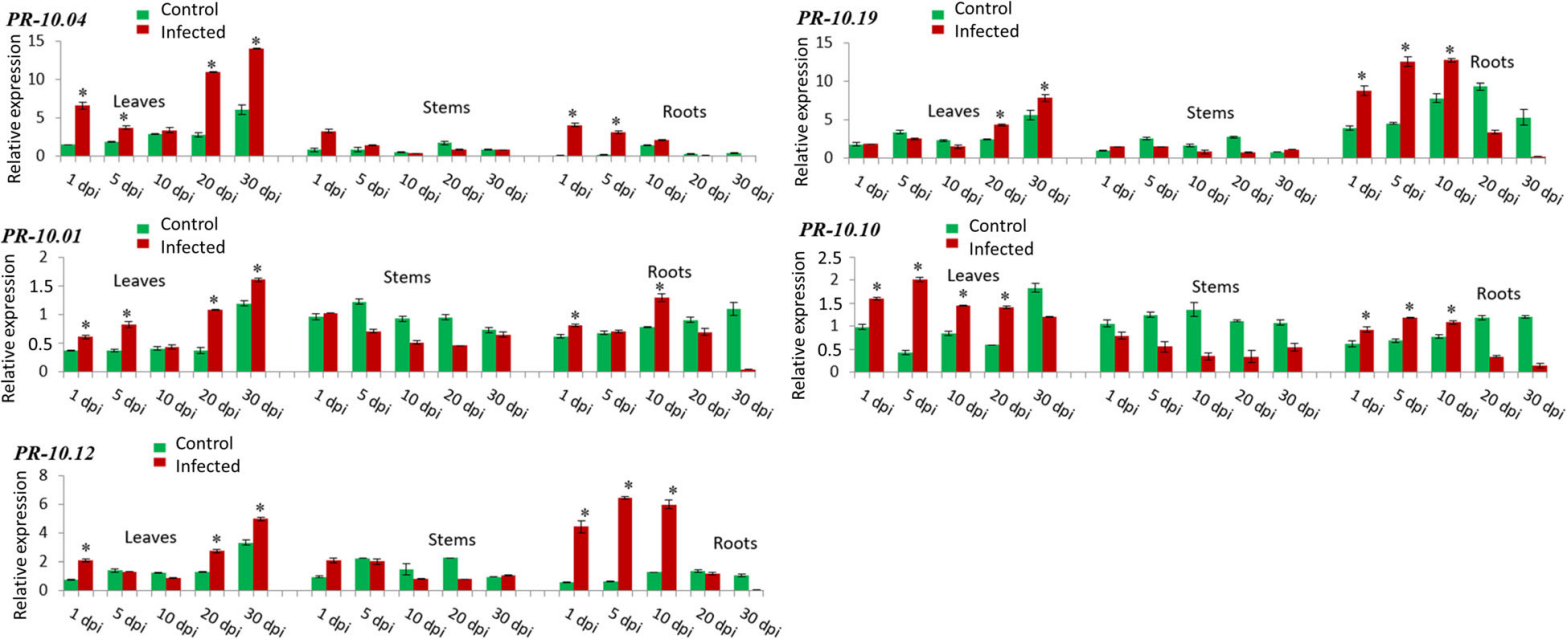
Relative transcript levels determined by qPCR of *PR-10.04*, *PR-10.01*, *PR-10.12*, *PR-10.19* and *PR-10.10* which are highly induced in leaves and roots. Strawberry plants were infected with *V. dahliae* and the relative transcript level was normalized to the control stem at 1 dpi (set to one). The expression levels in the tissues were measured at 1, 5, 10, 20, and 30 dpi (days post infection). Green bars represent the control while red bars represent infected tissues. The mean values (± SD) were obtained from five biological replicates and are shown as relative changes. Asterisks represent significant induction of a gene after statistical comparison with the transcript abundance of the control sample using the Tukey test. *FaRIB413* was used as a housekeeping gene for normalization

Furthermore, *PR-10.04*, *PR-10.01*, *PR-10.12, PR-10.19* and *PR-10.10* were strongly induced in leaves and roots simultaneously (Fig. 4). For the first three genes, the induction was more relevant in leaves after 20 dpi. There was an up to 14.0 fold rel. Exp. at 30 dpi for *PR-10.04*, 1.6 fold rel. Exp. at 30 dpi for *PR-10.01* and 5.0 fold rel. Exp. also at 30 dpi for *PR-10.12* in leaves. In roots, an upregulation was detected at day 1 for *PR-10.04* (4.1 fold rel. Exp.) followed by a decrease until day 30, whereas a strong induction of expression in infected roots was observed after 10 days for *PR-10.01* (1.3 fold rel. Exp.) and 5 days for *PR-10.12* (6.4 fold rel. Exp.). For *PR-10.19*, there was a high expression level in leaves namely at day 20 (4.3 fold rel. Exp.) and day 30 (7.9 fold rel. Exp.) post pathogen inoculation while in roots a progressive induction was detected until the 10th day (12.7 fold rel. Exp.) followed directly by a severe decline from the 20th day (3.4 fold rel. Exp.) of infection. *PR-10.10* showed also a considerable upregulation in leaves at the first 5 days of infection (2.0 fold rel. Exp.) but this increase gradually diminished in the course of time. In roots, this gene started to be induced from day 1 (0.9 fold rel. Exp.) until day 10 (1.0 fold rel. Exp.) followed by a drastic decrease at day 30 (0.1 fold rel. Exp.) (Fig. 4).

**Fig. 5:**
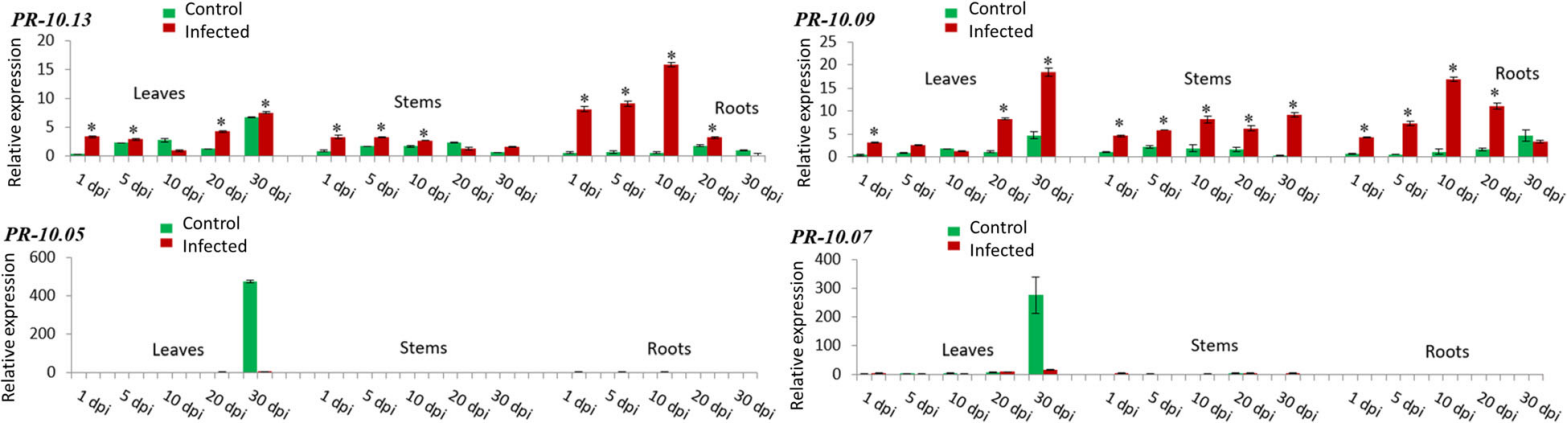
Relative transcript levels determined by qPCR of PR-10.13 and PR-10.09, which are highly induced in leaves, roots, and stems and relative transcript levels of PR-10.05 and PR-10.07 which indicated no induction for both isoforms. Strawberry plants were infected with *V. dahliae* and the relative transcript level was normalized to the control stem at 1 dpi (set to one). The expression levels in the tissues were measured at 1, 5, 10, 20, and 30 dpi (days post infection). Green bars represent the control while red bars represent infected tissues. The mean values (± SD) were obtained from five biological replicates and are shown as relative changes. Asterisks represent significant induction of a gene after statistical comparison with the transcript abundance of the control sample using the Tukey test. FaRIB413 was used as a housekeeping gene for normalization

Interestingly, the expression of *PR-10.13* and *PR-10.09* was induced in all three tissues at the same time (Fig. 5). In leaves, the expression pattern of both genes was very similar. It decreased progressively during the first 10 days post inoculation and augmented again until the 30th day (7.5 fold rel. Exp. for *PR-10.13* and 18.4 fold rel. Exp. for *PR-10.09*). In addition, those two genes were identically induced in roots. A gradual upregulation was observed until the 10th day of infection (15.8 fold rel. Exp. for *PR-10.13* and 17 fold rel. Exp. for *PR-10.09*) followed by a progressive diminution of expression until day 30 (0.2 fold rel. Exp. for *PR-10.13* and 3.3 fold rel. Exp. for *PR-10.09*). Moreover*, PR-10.13* and *PR-10.09* were the only two genes which were induced in stems. A steady decrease in the gene expression was detected for *PR-10.13* from the first day (3.3 fold rel. Exp.) and until the 30th day while for *PR-10.09* there were two peaks of expression, one peak at the 10th day (8.2 fold rel. Exp.) and the second peak at the 30th day (9.2 fold rel. Exp.) (Fig. 5). Interestingly, no induction of gene expression was detected in infected tissues for *PR-10.05* and *PR-10.07* (Fig. 5).

Altogether, a huge variation in transcript levels of the 21 isoforms was observed in the investigated *F*. *× ananassa* organs (leaves, stems and roots). The qPCR analysis indicated a clear upregulation of 19 isoforms after *Verticillium* infection and a different pattern of expression depending upon the analyzed strawberry tissue. The highest levels of induction were detected at later time point for leaves (20 to 30 days post pathogen inoculation) and early stage in roots (first 10 days post pathogen inoculation) **(**Additional file 4**)**.

### Liquid chromatography-mass spectrometry (LC-MS) analysis

**Fig. 6:**
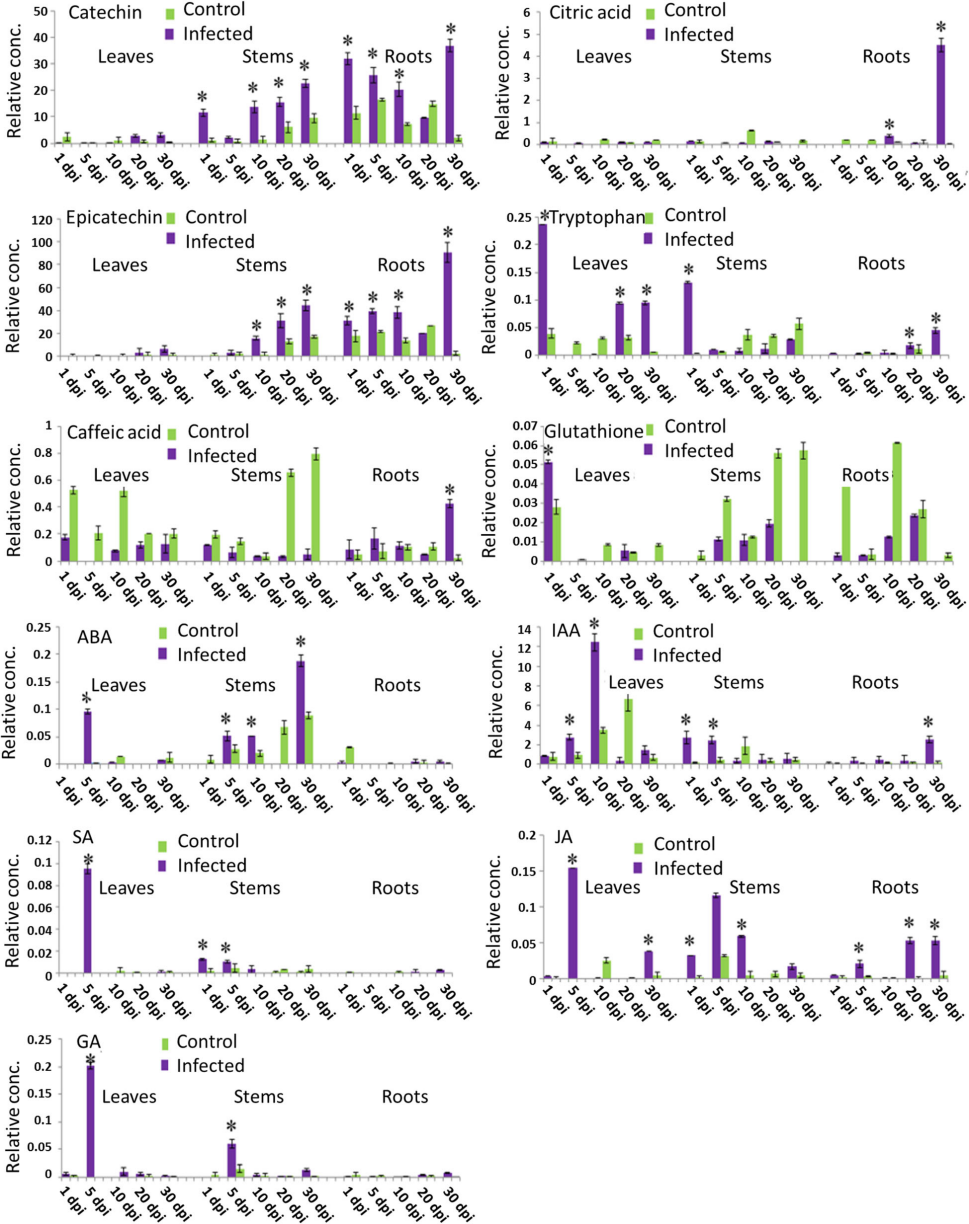
Relative concentration (± SD) of metabolites and pytohormones in infected (with *V. dahliae*) and uninfected *Fragaria* tissues at 1, 5, 10, 20, and 30 dpi (days post infection). Relative concentrations (Rel. conc.) are expressed in mg-equ./g dry weight of the internal standard biochanin A in freeze-dried strawberry tissues (leaves, stems and roots). Asterisks (*) indicate significant differences for each metabolite after statistical comparison with its abundance in the control sample using the Tukey test

In order to test whether plant metabolites are involved in the defense response toward *Verticillium* infection in strawberry in vitro plants, tissues used for qPCR were also analyzed by LC-MS (Fig. 6). Metabolites such as catechin and epicatechin accumulated in stems and roots over time in infected tissues and exceeded the levels in the control plants considerably. Caffeic acid and citric acid were observed mainly in infected roots at 30 dpi. Tryptophan was observed in infected leaves and stems at 1 dpi as well as at late stage in infected leaves and roots (20–30 dpi). Glutathione in its reduced form (GSH) was mainly detected in control stems and roots and at 1 dpi in infected leaves. Furthermore, the implication of some phytohormones on strawberry defense was also tested. Five plant hormones were identified by LC-MS. Abscisic acid (ABA), salicylic acid (SA), jasmonic acid (JA), and gibberellic acid (GA) accumulated in infected leaves at 5 dpi and at 10 dpi for indole-3-acetic acid (IAA). The five phytohormones were also detected in infected stems at various time points: during the first 5 dpi for SA, IAA and GA, and during the first 10 days post inoculation for JA. Likewise, ABA was identified during the first 10 days in infected stems with a peak at 30 dpi. IAA and JA were the unique hormones to be discerned in infected roots at tardy stages (20 and 30 dpi) (Fig. 6).

## Discussion

### *PR-10* gene expression

The present study clearly demonstrated that the expression of various *PR-10* genes was induced by *Verticillium* infection in in vitro strawberry plants. *PR-10.03*, *PR-10.11, PR-10.16* and *PR-10.21* were significantly induced in leaves (Fig. 2). The gene expression levels peaked in leaves 20–30 dpi for *PR-10.03* and *PR-10.16* and from 1 to 5 dpi for *PR-10.21* and *PR-10.11*. Apoplastic proteome analysis in leaves of oilseed rape infected with *V. longisporum* revealed the up-regulation of PR-2 (β-1,3-glucanase), PR-3 (chitinase) and PR-4 (chitinase) [20]. In lettuce infected with *V. dahliae*, *PR-3* and *PR-5* (thaumatin-like) genes were expressed only in symptomatic leaves harvested 3 weeks after infection [21]. Thus, infection by *Verticillium* caused the up-regulation of multiple PR genes in leaves including *PR-2*, *PR-3*, *PR-4* [20], *PR-5* [21], and *PR-10* [this study].

Transcription of *PR-10.02, PR-10.06, PR-10.08*, *PR-10.14, PR-10.15, PR-10.17, PR-10.18* and *PR-10.20* was strongly induced in roots upon *Verticillium* infection. Notably, *PR-10.17* was exclusively transcribed in roots with the highest expression value within the 21 *PR-10* isoforms (Fig. 3). The upregulation was mainly observed during the first 10 days post inoculation. Although plant roots are in close contact with a diversity of soil microorganisms, interactions between them are still poorly studied [22, 23]. Recently, *Arabidopsis thaliana* was used as a host model for *V. dahliae* [24, 25] and *V. longisporum* [26] to analyze the interaction between roots and vascular pathogens. In *A. thaliana* roots infected with *V. longisporum*, genes involved in the production of tryptophan-derived secondary metabolites showed the highest transcript levels at 8 dpi [27]. Similarly, in our study, a peak of expression in roots was reached around 10 dpi suggesting that the fungus is spreading over time until a decline of expression is reached at 30 dpi. This decrease is probably due to plant resistant responses followed by fungus elimination around 30 dpi. Pectinaceous gels, formed in xylem vessels of plants infected with *Fusarium oxysporum*, are considered an effective defense system, as pathogen colonization of the upper parts of stem and leaves is blocked. The pathogen remains in the infected roots and cannot spread [28]. Thus, according to this study and all the cited results, the high level of some PR-10 proteins in roots is probably part of a constitutive defense mechanism in this plant organ that is most exposed to environmental stress.

Interestingly, *PR-10.01*, *PR-10.04*, *PR-10.10, PR-10.12* and *PR-10.19* isoforms were considerably expressed in both roots (early stage) and leaves (late stage) and hence correlated with the time course of infestation (Fig. 4). This finding is also in accordance with the induction of *PR-10c* in leaves and roots of birch in response to oxidative stress [29]. Overall, the expression levels of the 17 above-cited *PR-10* genes revealed that leaves and roots differ considerably in executing their temporal defense responses.

A striking expression was observed for *PR-10.13* and *PR-10.09,* which were induced in all three tissues (leaves, roots and stems) simultaneously (Fig. 5). Several studies showed that *Verticillium* species enter the xylem vessels of the root and start sporulating after 2 to 5 dpi followed by a colonization of stem vessels few days later [11, 30]. In *Verticillium*-infected hop stems [31], the fungal biomass increased through time in accordance with the transcript level of *PR-10.09* in stems while the expression of *PR-10.13* decreased steadily until 30 dpi. Also in stems of tomato (*Solanum lycopersicum*) infected with *V. dahliae*, the expression pattern of two *PR*-genes *Ve1* and *Ve2* encoding cell surface receptor proteins of the extracellular leucine-rich repeat receptor-like protein class of disease resistance proteins revealed that both genes exhibited high transcript levels 10–12 dpi in the susceptible isolines [32]. *In Verticillium*-infected tomatoes, the host compatibility (susceptibility) or incompatibility (resistance) with *Verticillium* appeared to be determined in stems [33, 34]. Moreover, during *Verticillium* colonization in this tissue, fungal elimination predominates because the resistant cultivar plant is able to activate defense responses faster [30, 35] while in the susceptible cultivar the pathogen escapes from this elimination and spreads inside the plants vascular system [11, 36].

In strawberry, both resistance and tolerance can enhance the global performance of different genotypes in the presence of *V. dahliae* but tolerance may be less stable over the course of a season [37]. Contrary to plant disease resistance, which protect plants from pathogen attack, plant disease tolerance is a specific phenomenon that includes down-regulation of genes associated with development of symptoms [38]. Most of the 41 evaluated strawberry genotypes from the University of California breeding program were identified as highly susceptible to *V. dahliae* however, eleven strawberry (*F. × ananassa*) genotypes were designated as resistant to *Verticillium* wilt [37, 39]. Strawberry resistance to a variety of pathogens has been bred into commercial varieties as a polygenic trait and has been reported to be quantitatively inherited [40]. The nonappearance of symptoms despite high levels of *V. dahliae* inoculum could result from a decreased incidence of systemic infection and from reduced symptom expression after pathogen entry into the xylem. The roles of *PR*-genes in the *V. dahliae* resistant genotypes has not yet been well studied.

In the present work, after *Verticillium* inoculation, a visual assessment of the foliar symptoms of *F. × ananassa* in vitro plants was performed. The scale employed for the visual assessment of the disease was based on the percentage of symptomatic leaf area. The strawberry plants seemed to be “healthy” during the first 20 dpi (class 0) since no visible foliar wilting symptoms were observed. The diffusion of strawberry leaf symptoms began with class 2 equivalent to 10–25% of symptomatic leaf area at 20 dpi and only after 10 additional days, the fungus was able to colonize almost the totality (75–100%) of the leaf surface producing necrotic leaves, which were sometimes stunted in stature. The disease severity was considerable beyond 36 dpi as the in vitro plants faded completely resulting in wilting and plant death, which explained consequently, why plant material for gene expression analysis was not more recoverable after this time point. Similarly, the response to the infection caused by *V. dahliae* in two micropropagated *F. × ananassa* cultivars became evident only after 15 dpi and, differently to our results, the highest percentage of totally chlorotic microplants was obtained at 75 dpi for both subclones ‘Filon’ and ‘Teresa’ [41]. While our findings clearly demonstrated an initiation of transcriptional responses in leaves, roots and stems during invasion by the *Verticillium* fungus for nineteen of the *PR-10* genes, *PR-10.05* and *PR-10.07* seemed to escape from the fungus colonization since no induction was detected in any tissue for both genes.

Studying defense gene expression implies the use of a suitable host-pathogen model system and respective culture conditions. In strawberry, many researchers have applied in vitro screening systems to obtain plants resistant or tolerant to *Rhizoctonia fragariae* and *Botrytis cinerea* [42], *Colletotrichum acutatum* [43], *Fusarium oxysporum* [44], *Phytophthora cactorum, P. fragariae* [45] and *V. dahliae* [46]. In vitro selection, the technique used in this work, is a useful tool in identifying plants resistant or tolerant to stresses produced by phytotoxins from pathogens, cold temperature and salt toxicity [47]. Susceptibility of strawberry cultivars and breeding lines to infection by *V. dahliae* under in vitro conditions was similar to their response to infection in field conditions [48].

### Tissues analysis by LC-UV-ESI-MS

#### Secondary metabolites

Flavan-3-ols such as catechin and epicatechin accumulated in infected stems and roots especially at late stages after infection by the fungus *V. dahliae* (Fig. 6). This result suggested that increased production of catechin and epicatechin during *Verticillium* inoculation exercise a protective role in suppressing fungal infection. (+)-Catechin, which already pre-existed in strawberry leaves, inhibited *Alternaria alternate* and induction of resistance response in the strawberry leaf to this fungus provoked the accumulation of (+)-catechin [49]. Accumulation of catechin-derived procyanidins was fundamental to inhibit the growth of *Botrytis cinerea* in immature strawberry fruits [50]. Moreover, *in Centaurea maculosa* or spotted knapweed, (±)-catechin has been reported to be released from its roots [51] showing an inhibitory effect on soil microbial activity [52]. Likewise, Veluri et al. [53] reported an antimicrobial and antifungal effect of (+)-catechin to some soil strains (*Xanthomonas campestris, Agrobacterium radiobacter, Erwinia carotovora,* and *Erwinia amylovora*). Such activities of (+)-catechin on certain soil microbial isolates were not detected for the (−)-enantiomer of catechin, which was revealed to be phytotoxic [54]. Besides, it has been reported that epicatechin was able to inhibit lipoxygenase from *Colletotrichum gloeosporioides*, which inactivated antifungal dienes (1-acetoxy-2-hydroxy-4-oxo-heneicosa-12, 15-diene) formed during the ripening of avocado [55]. The flavan-3-ol, which is present in unripe fruit at high concentration, might also be involved in the resistance of avocado fruits by inhibiting the pectate lyase activity of *C. gloeosporioides* [56]. The flavan-3-ols catechin and epicatechin have also anti-oxidative and radical-scavenging activities that exceed those of ascorbic acid, α-tocopherol and other phenolics [57].

Contrary to the caffeic acid levels, which significantly decreased in tomato roots upon *Arbuscular mycorrhizal* colonization [58], levels of caffeic acid were induced in *F. × ananassa* infected roots particularly after 30 dpi in this study. Caffeic acid, which plays a role in plant defense reactions, showed strong in vitro antimicrobial activity against numerous fungi including *V. dahliae*. It has been shown that this antioxidant compound is increased in *Verticillium*-infected cotton plants [59] and in potato with a differential accumulation in roots and stems [60]. In pepper roots, the fungus *Fusarium oxysporum* stimulated the biosynthesis of caffeic acid and primed that of chlorogenic acid [61].

Similarly, accumulation of citric acid was induced at late stage in infected roots (30 dpi) supporting the hypothesis that this acid affects growth and health of the plant host. Root secretion of citric acid was enhanced in *Fusarium oxysporum f. sp. cucumerinum*-infected cucumber plants and citric acid acted as a chemoattractant in this process [62]. Root exudates from tomato, cucumber and sweet pepper plants grown under gnotobiotic conditions contained higher total amounts of organic acids (citric acid) than of carbohydrates [63]. The rhizobial microflora affects the composition of root exudates as application of the bacterial biocontrol strain *Pseudomonas fluorescens* to tomato roots led to reduced levels of succinic acid, whereas the concentration of citric acid increased [64].

In this work, the levels of tryptophan, another strawberry plant constituent, in infected tissues exceeded the level in control plants after 1 dpi in leaves and stems, and 20–30 dpi in roots. It appears that strawberry plants have evolved various defense mechanisms against biotic stress, including the production of primary and secondary metabolites, which act as defense compounds. Leaves of *F. × ananassa* inoculated with the angular leaf spot bacterium, *Xanthomonas fragariae*, produced high level of tryptophan, which is a precursor of aromatic secondary metabolites [65]. The tryptophan pathway plays also an important role in the defense responses against pathogenic infection in *Arabidopsis* root [27] as well as in rice [66].

The accumulation of the reduced form of glutathione upon fungal infection was exclusively observed in this work in infected leaves after 1 dpi revealing a quick response to *Verticillium* infection. Glutathione probably plays a role in the plant cells response to physical as well as biological stresses. Glutathione is a major cellular antioxidant and both glutathione metabolism and levels influence cellular defenses [67]. In most plant tissues, glutathione is predominantly present in its reduced form (GSH) but the oxidized disulphide form of glutathione (GSSG) can also be detected. GSH and its homologues are considered as essential components in plant cell by participating in the elimination of reactive oxygen species (ROS) via thiol-disulphide redox reactions and in detoxification of various xenobiotics by conjugation reactions [68, 69]. GSH is involved in response to oxidative and abiotic stress, which rapidly accumulated after fungal attack [70]. Participation of GSH in antioxidative defense reactions and detoxification appeared to be the principal role of GSH in plant tissues either by direct reactions with reactive oxygen species or through the ascorbate-GSH cycle [70]. Moreover, both GSH and GSSG elicited the phytoalexin response in cell-suspension cultures of bean but had no effect in alfalfa [71]. A decline in GSH content in oat leaves infected by the fungal pathogen *Drechslera* approximately 2 days post-inoculation indicatied an increased demand for detoxification of reactive oxygen species in the early stages of pathogenesis [72]. Similarly, except for a high level of GSH at 1 dpi in infected leaves, lower levels of GSH were detected in this work in infected tissues in comparison with the controls leading. This led to the conclusion that the infected leaf tissue showed a reduced capacity to resist oxidative stress and to repair damage. The changes in GSH level was also quantified in tomato leaves carrying either the genes Cf*-2* or Cf*-9* conferring resistance against the fungal pathogen *Cladosporium fulvum* [73]. The total glutathione level increased 2 to 8 h after the injection of elicitor into the leaves and that 87% of this accumulation was attributed to the GSSG form. Significant increases in the foliar GSH levels were also observed in different oat lines (*Avena sativa*) 24 h after infection with *B. graminis f. sp. Avenae* [74] and in resistant tobacco (*Nicotiana tabacum L cv. Xanthi-nc*) plants infected with Tobacco Mosaic Virus (TMV) [75].

#### Phytohormones

Phytohormones such as ABA, SA, GA, IAA and JA are major signaling molecules in plants during stress response and their levels have been investigated in *Verticillium* infected *F. × ananassa* in vitro plants. A critical step in plant defense is the sensing of the stress in order to respond in a quick and efficient manner. The perception of abiotic and biotic stress conditions initiates signaling cascades that open or close ion channels, activate or inactivate kinases, produce reactive oxygen species (ROS) and lead to the production of phytohormones.

In the present study, the ABA concentration in infected tissues exceeded the level in controls in leaves at 5 dpi and in stems during the first 10 dpi with a peak at 30 post inoculation revealing a crucial role in defense response. Such findings suggested that the ABA signaling pathway is a candidate for the control of systemic colonization by *V. dahliae* in *strawberry.* ABA has a prominent role during pathogen infection [76, 77], or is just involved in the perception of environmental stresses, particularly osmotic stresses [78]. The concentration of ABA increased in sugar beet leaves during *Cercospora beticola* fungus infection and elevated ABA concentrations were detected during colonization at 3 to 9 dpi [79]. The role of ABA in disease resistance is discussed controversial as it depends on numerous factors, e.g. the type of the attacking pathogen, its entry point and way into the host, the timing of the defense response, and the plant tissue that is attacked by the pathogen. Thus, ABA has multiple effects depending on the environmental conditions and its exact role in plant pathogen interaction is still a matter of debate.

Another major hormone produced by plants is gibberellic acid (GA), which is also known to participate mainly in abiotic stress [80]. In the nineteenth century, GA was identified as being responsible for the excessive growth of rice seeds infected with the fungal pathogen *Gibberella fujikuroi* in Japan and only recently GA has been considered to be implicated in plant responses to pathogen attack and the role of GA in plant immunity has been studied. The outer capsid protein P2 of the rice dwarf virus (RDV) interacted with plant ent-kaurene oxidases, affecting the production of GA and the RDV infected rice plants showed a significant reduction in GA [81]. Those finding are consistent with the present study, which revealed that GA accumulated in comparison with non-infected tissues at 5 dpi in leaves and stems and decreased during all the following infection days while in infected roots no significant GA level was detected during the 30 days of *Verticillium* infection. Thus, GA is part of the hormonal system used by plants in defense signaling pathways during pathogen colonization. The production of gibberellin by *Verticillium* species using dwarf mutant seedlings of *Zea mays* was investigated [82]. From 114 isolates of *Verticillium* grown on potato-carrot dextrose medium for 15 to 25 days, only 27 isolates produced “gibberellins” and 12 were identified as *V. dahliae*. Besides, treatment of sunflower with GA was able to prevent the stunting of plants seedlings inoculated with *Verticillium*, indicating that it may result from interference with normal GA activity. GA promotes degradation of DELLA proteins, which are negative regulators of growth and thus GA stimulates plant growth. In addition, DELLA proteins, which act also as negative regulators of GA signaling in *Arabidopsis*, modulate SA and JA dependent defense responses [83]. An *Arabidopsis* quadruple-DELLA mutant that lacked four *DELLA* genes was resistant to the biotrophic pathogens Pto DC3000 and *Hyaloperonospora arabidopsis* [83] but very susceptible to *Botrytis cinerea*, a necrotrophic fungus. Besides, GA is synthesized by the necrotrophic pathogens *G. fujikuroi* as a virulence factor to degrade DELLA proteins and to confer host susceptibility. In this case, GA is not only interfering with SA/JA-dependent defenses but also by interfering the ROS balance and leading to death. Thus, the mechanism of action of GA in response to pathogen attack is still a topic of debate.

While ABA and GA are predominantly involved in abiotic stress, SA and JA are more responsible for the plant’s reaction to biotic stress. In this study, SA accumulated in comparison with non-infected strawberry plants exclusively in leaves at 5 dpi whereas JA exceeded the levels in the same infected tissue at 5 and 30 dpi. In stems, JA accumulated, in comparison with non-infected plants during the first 10 days post *Verticillium* infection and SA during the first 5 days while in infected roots only JA was identified at late stages (20–30 dpi). These results clearly demonstrate that both SA and JA defense signaling pathways are activated in strawberry plants during *Verticillium* colonization. The contents of SA and JA in *F. × ananassa* cv. Camarosa plantlets infected with *Colletotrichum acutatum* was studied [84]. The most significant SA and JA concentration in aerial tissues was also obtained after 5 dpi. A number of known components of both SA and JA-dependent defense pathways such as *FaPR1–1* and *FaGST1* (oxidative stress-responsive defense genes) were not significantly accumulated in strawberry during interaction with *C. acutatum.* Thus, only specific branches of both SA and JA-dependent defense pathways are affected by this pathogen in strawberry plants supporting the idea that this pathogen uses a molecular strategy to bypass the plant defense. Plants have complex defense mechanisms to withstand microbial and fungal pathogen attacks and hypersensitivity. The most powerful mode of resistance against pathogen attack is the accumulation of SA and certain PR proteins [85]. The xylem sap from hypocotyl and root of *Brassica napus* infected with *V. longisporum* contained high levels of SA that correlated to the amount of pathogen DNA and the degree of stunting [86]. SA is an import plant hormone implicated in the protection of plants against biotrophic and hemibiotrophic pathogens and plays a role in the establishment of systemic acquired resistance (SAR) [87]. Thus, SA is also involved in defense responses of plant tissues against *Verticillium* toxins [88]. In contrast, JA usually mediates protection against herbivorous insects and necrotrophic pathogens [89]. *Verticillium* species are hemibiotroph plant pathogenic fungi showing a necrotrophic phase during the late stages of infection. Therefore and as confirmed in this work, the plant SA defense signaling pathway is activated during the first biotrophic stage of *Verticillium* infection while the JA signaling pathway is activated in leaves, stems and roots also at the late phases. Briefly, the success of pathogen infection and growth depends on the balance of how fast the pathogen escapes the plant defenses and how quick the plant adapts the defense response. Besides, possible manipulations of the host’s hormone status by the pathogen for its own benefit should be considered.

Because of the availability of suitable mutants in *Arabidopsis thaliana*, most of the experiments on phytohormones were conducted in this plant. One of the important regulatory components of SA signaling is the non-expressor of PR genes 1 (NPR1), which interacts with TGA (TGACG motif-binding protein family) transcription factors that are involved in the activation of SA-responsive PR genes [90]. Downstream of NPR1, several WRKY transcription factors (containing one or two WRKY protein domains) play important roles in the regulation of SA-dependent defense responses in plants [91, 92].

Interestingly, a rapid accumulation of JA during the first 11 h post inoculation (hpi), but not of SA, in phloem exudates of leaves challenged with an avirulent strain of *Pseudomonas syringae* and increased expression levels of JA biosynthetic genes suggested that JA could act as a mobile signal in *Arabidopsis* pathogen immunity [93]. Likewise, other researchers established that concentrations of JA increase in response to pathogen infection or tissue damage and that the expression of defense related genes is induced [94, 95]. Gene expression in response to JA is modulated among others by a transcription factor JA insensitive 1/MYC2 (JIN1/MYC2) [96], members of the Apetala2/ethylene-responsive factor (AP2/ERF) family [97], an F-box coronatine insensitive protein 1 (COI1) [98], a plant defensin protein 1.2 (PDF 1.2) [99] and a repressor Jasmonate-ZIM-Domain protein (JAZ) [100]. A critical regulator mediating plant protection during *V. dahliae* infection appears to be a GbWRKY1 transcription factor, which activated JAZ1 expression in cotton [101]. Furthermore, the interaction between defense signaling pathways of SA and JA is an important mechanism for regulating defense responses against various types of pathogens. The *Arabidopsis* WRKY70 (transcription factor) [102, 103], MPK4 (Mitogen activated protein kinase 4) [104] and GRX480 (glutaredoxin) [105] have been found to regulate the antagonistic interaction between SA and JA-mediated defenses. In this work, JA levels in roots increased at late stages and those of SA were insignificant at this phase. This finding suggests an antagonistic relationship between the two pathways in strawberry. In addition, evidence of synergistic interactions between SA and JA defense pathways has been reported. MeJA and SA induced the transcription factor WRKY62 synergistically after pathogen inoculation [106] and activated the expression of some defense related genes [107].

Besides the aforementioned phytohormones, IAA was also analyzed in this work. Significant levels of IAA were detected in all infected samples. In leaves, IAA concentration increased gradually in comparison with the non-infected controls with a peak at 10 dpi and then decreased until 30 dpi. IAA accumulated in comparison with the controls in stems during the first 5 days post *Verticillium* infection and exclusively in roots at 30 dpi. Such results suggested that IAA played also a crucial role in strawberry defense response toward *Verticllium* infection. A significant increase in IAA accumulation was also demonstrated in plant leaves infected with either Xcc (*Arabidopsis*–*Xanthomonas campestris* pv. *campestris*) or Pto (*Pseudomonas syringae* pv. *tomato*) with a maximal level occurring 3 to 4 days after infection [108]. Auxin responsive *GH3* genes to play roles in the protection of *Arabidopsis* and rice. *GH3.5* in *Arabidopsis*, is a bifunctional modulator during pathogen infection acting in both SA and auxin signaling [109]. In rice, overexpression of *GH3.8* promoted resistance to the pathogen *Xanthomonas oryzae* pv *oryzae* (Xoo) in a SA and JA independent pathway [110]. *GH3–8* impeded IAA-induced expansin production, which is probably reasonable for the resistance against *Xoo*. One of the mechanisms used by Xoo to infect plants *is the* IAA-stimulated local production of the expansin protein. In addition, IAA and SA interact antagonistically, thereby enhancing susceptibility to biotrophic pathogens [111]. Interestingly, transport inhibitor response protein 1 (TIR1), whose expression is repressed by SA, is an IAA receptor and interacts with Aux/IAA proteins [112, 113].

This work represents the first study of the hormonal implication in strawberry *F. × ananassa* in response to *Verticillium* infection. Nevertheless, the fundamental molecular mechanisms and the role of those compounds toward pathogen attack in strawberry are not fully understood. How precisely the machinery of phytohormone signaling differs between *Verticillium* strains in strawberry, how does the plant modulate the level of phytohormones in response to numerous simultaneous pathogens attacks including *Verticillium* and which mechanisms may underlie a suitable control of signaling response to *Verticillium* infection, remain challenging questions for future investigations.

## Conclusions

The gene expression of the twenty-one *PR-10* genes revealed strong variation in transcript levels and a wide range of response. Nineteen *PR-10* genes were clearly upregulated after *Verticillium* infection. The highest levels of expression were detected at early stage in roots and later time point for leaves consistent with the colonization of the plant. Analysis by LC-MS showed an accumulation of phytohormones in leaves, stems and roots already at 5 dpi demonstrating a fast response of the *F. × ananassa* plants after pathogen infection and providing evidence of the involvement of phytohormones in plant defense signaling pathways. Antifungal metabolites accumulated at later periods after *Verticillium* infection. The work nicely illustrates the different defense strategies of the strawberry plant against attack by fungi.

## Methods

### Propagation of in vitro plants

For the propagation of *F. × ananassa* cv. Elsanta in vitro plants, a revised Murashige and Skoog (MS) medium was used [114] and 20 g/l of sucrose and 6.5 g/l of agar were added. After adjusting the pH to 5.8 by HCl or KOH (1 M), the medium was autoclaved at 121 °C for 20 min. After cooling down the agar to 45–50 °C, the medium was supplemented with 50 mg/l of citric acid and ascorbic acid and finally dispensed (30 g) in 200-ml-jars. Once the agar was solidified, the in vitro plants were transferred into the jars and the strawberry culture was maintained for around 6 weeks. *F. × ananassa* in vitro plants were grown in a dedicated room with a 16 h photoperiod. The average of the daytime temperature was 23.8 ± 1.4 °C while during the night the temperature was around 22.0 ± 1.5 °C.

### Infection with *Verticillium*

*F. × ananassa* in vitro plants were artificially inoculated by the isolate E650 of *V. dahliae*, kindly provided by the Institute for Breeding Research on Horticultural Crops, Dresden. This isolate caused a weak to medium damage to the *F. × ananassa* cultivar used for this work*. V. dahliae* was cultured on potato dextrose agar using two layers of gauzes. The petri dishes were stored in the darkness for 3–4 weeks at 22 °C (Additional file 1). One month later, the gauzes were immerged and swirled in 10 ml of sterile distilled water. After filtration to remove hyphae, the conidia concentration was adjusted to 10^6^ spores/ml using a Thoma hemocytometer. In vitro plants were inoculated directly on the agar medium by piercing the roots with the pipette tip at five different locations (10^6^ conidia/ml).

Control plants were similarly inoculated with sterile water. Plants were kept in a growing chamber, harvested at 1, 5, 10, 20 and 30 dpi and separated into leaves, stems, and roots. Five infected plants and five controls were taken at each time point. Leaf, root and stem samples were immediately frozen in liquid nitrogen and stored at − 80 °C. At all sampling time points, plants were visually assessed for the appearance of foliar symptoms using a 0–5 scale according to [9].

### Primer design

Specific primers were designed and verified using different bioinformatics tools (BioEdit/ Clustal 2.1/ Primer3/ OligoAnalyzer 3.1). Primer3 has many different input parameters, which can be controlled to define characteristics that allow the software to design primers suitable for qPCR analysis. For SYBR Green-based qPCR primer design, the following parameters were chosen: primer length of 18–30 base pairs (bp), primer melting temperature (Tm) between 58 °C and 64 °C, and guanine-cytosine (GC) content of 20–80%. Other criteria were the product size of less than 200 bp and prevention of repeats in the nucleotide sequences to avoid mispriming (e.g. ACACACACAC). Specific primers for the 21 *PR-10* isoforms are listed in Additional file 3. In order to check the gene specificity and to verify that the primers do not hybridize with another gene, each primer pair was blasted using in silico analysis. This was done by comparing the primer sequences to known gene databases, using the BLAST option. In addition, amplification products were subjected to melting curve analysis and single peaks indicated specificity.

### RNA extraction

Total RNA was isolated as described [115] using the CTAB method. A 20 ml aliquot of pre-heated extraction buffer was added to 1–2 g of lyophilized strawberry material. During the incubation of the mixture at 65 °C, Falcon tubes were inverted manually 3 times. Then, an equal volume of chloroform/isoamyl alcohol was added and the samples were again gently inverted by hand for 10 min. After centrifugation at 12,000 rpm (4 °C), the supernatant was transferred to a new Falcon tube and a 1/3 volume of 8 M LiCl was added to the reaction. RNA precipitation was carried out overnight at 4 °C. After centrifugation at 12,000 rpm, for 30 min at 4 °C, the RNA pellet was gently dissolved in 500 μl 0.5% SDS. A second centrifugation step was following and the supernatant was then transferred to a new Eppendorf tube containing two volumes of 100% ethanol. The reaction was performed for 2 h at − 20 °C. After centrifugation of the precipitated RNA (30 min, 4 °C, 13,200 rpm), the pellet was washed with 70% and the 100% ethanol. Subsequently, the pellet was air-dried and then dissolved in 50 μl DEPC. The quality and quantity of RNA was determined using Nanodrop and 1% agarose gel.

### Synthesis of cDNA

One μg of DNase-treated RNA was reverse transcribed to cDNA using 1 μl of random primers (10 μM) and 1 μl of 10 mM dNTP in 20 μl total volume. After a five minutes incubation time at 65 °C, the reaction mix was chilled on ice for 1 min. A First strand buffer and RNase OUT recombinant RNase inhibitor were added to a nuclease-free-micro centrifuge tube. Later, one μl of M-MLV RT was added after an incubation of 2 min at 37 °C. An incubation step at 25 °C for 10 min was performed before synthesizing cDNA at 50 °C for one hour. Then, the temperature was raised to 70 °C to inactivate the reverse transcriptase and the cDNA was stored at − 20 °C.

### Quantitative real time PCR (qPCR)

Quantitative real time PCR analyses were performed using the StepOne Realtime PCR Instrument and Software (Applied Biosystems, Darmstadt, Germany). A Master Mix for each PCR run was prepared with SYBR Green (Applied Biosystems). The amplification was carried out as follows in 20 μl total volume: 10 μl Fast SYBR Green Master Mix, 10 μM forward Primer, 10 μM reverse Primer and 2 μl of the template (cDNA). The PCR amplification program was optimized for 40 cycles using the following standard thermal program: 95 °C for 10 min, then 95 °C for 15 s and 60 °C for 1 min (40 cycles), followed by 95 °C for 15 s, 60 °C for 1 min and 95 °C for 15 s. The amplification was carried out in a 96-well reaction plate (Applied Biosystems) and all samples were amplified in at least three technical replicates. A cDNA dilution of 1/20 for the target gene and 1/8000 for the housekeeping gene were found enough sensitive for the gene quantification studies. The Ct values (threshold cycle value) of each *PR-10* gene were normalized to 1 dpi in control stems using the Ct value of the strawberry *FaRIB413* gene which is corresponding to an internal RNA interspacer (16S–23S). The fold change of gene expression 2^-ΔΔCt^ was calculated according to [116].

### LC-MS analysis

For this approach an internal standard, containing 50 mg of biochanin A and 50 mg 4-methylumbelliferyl-ß-D-glucuronide which were dissolved by sonication for 10 min in 250 ml methanol, was prepared. For the extraction procedure, 50 mg of lyophilized tissues (leaves, roots and stems) of infected and non-infected strawberry plants were mixed with 250 μl of internal standard and 250 μl of methanol. Samples were vortexed for 1 min, sonicated for 10 min and centrifuged also for 10 min at 13,200 rpm. Supernatant was taken into a new Eppendorf tube and the pellet was extracted again with 500 μl of methanol. Then, both supernatants were combined and treated with Speed-Vac System for 2–3 h to evaporate the methanol. The dried pellet was re-suspended in 35 μl LC-MS quality water, vortexed, sonicated and centrifuged twice at max speed. Finally, the clear supernatant was taken into an LC-MS vial with micro-insert and analyzed by LC-UV-ESI-MS. The accumulation of metabolites in leaves, stems and roots of infected and non-infected in vitro plants was tested referring to their retention time and fragmentation pattern by the means of QuantAnalysis software. This tool was used for quantification and results were normalized against the internal standard (biochanin A). The accumulation of the below mentioned metabolites is designed as the relative concentration (Rel. conc.) which is expressed in mg-equ./g dry weight of the internal standard biochanin A in freeze-dried strawberry tissues (leaves, stems and roots).

Abscisic acid (ABA, 264.32 g/mol), salicylic acid (SA, 138.12 g/mol), jasmonic acid (JA, 210.27 g/mol), gibberellic acid (GA, 346.47 g/mol) and indole acetic acid (IAA, 175.19 g/mol) were eluted at 37.9 min, 35.5 min, 39.1 min, 30.8 min and 33.3 min, respectively. The retention times were confirmed by analyses of authentical reference materials.

### Statistical analysis

The statistical analysis was carried out using the statistical program SAS (SAS Institute Inc., Cary, USA). A one-way analysis of variance (ANOVA) was performed for each inoculation day and the mean values of the relative expression level of the 21 genes were analyzed by the Tukey test. The significance level was *p* ≤ 0.05.

### Writing guideline

Symbols for gene names are denoted in italic (e.g., *PR-10.06*) while symbols for protein names are not italicized (e.g., PR-10.06).

## Additional files

Additional file 1: Culture of *Verticillium dahliae* in potato dextrose agar medium. The bottom (right) and the top (left) of the Petri dish represent the evolution of the culture after 3–4 weeks. (PPTX 548 kb)

Additional file 2: PR-10 nomenclature and gene number (PPTX 34 kb)

Additional file 3: Primers of the 21 *PR-10* isoforms used for qPCR analysis. (PPTX 44 kb)

Additional file 4: Phylogenetic tree of *PR-10* genes and summary of the expression profile of the 21 *PR-10* isoforms in untreated leaves, stems and roots of in vitro cultivated *F. × ananassa* plants (green bars) and infected tissues with *V. dahliae* (red bars) at various time points post (days) pathogen inoculation. qPCR using specific primers for *PR-10* genes and the reference gene was used for the expression analysis. (PPTX 238 kb)

## Abbreviations

ABA: Abscisic acid
BLAST: Basic Local Alignment Search Tool
dpi: Days post infection
GA: Gibberellic acid
GSH: Glutathione
GSSH: Oxidized glutathione
IAA: Indole acetic acid
JA: Jasmonic acid
LC-MS: Liquid chromatography-mass spectrometry
PR-10: Pathogenesis-related protein family 10
qPCR: Quantitative polymerase chain reaction
RDV: Rice dwarf virus
ROS: Reactive oxygen species
SA: Salicylic acid
Tm: Melting temperature
