# Supplementary material for: Induction of PR-10 genes and metabolites in strawberry plants in response to Verticillium dahliae infection
Source: BMC Plant Biol. 2019 Apr 5;19:128. doi: 10.1186/s12870-019-1718-x (PMC6451215; doi:10.1186/s12870-019-1718-x)
Supplement: Supplementary file 1 — Culture of Verticillium dahliae in potato dextrose agar medium. The bottom (right) and the top (left) of the Petri dish represent the evolution of the culture after 3–4 weeks. (PPTX 548 kb) [file 12870_2019_1718_MOESM1_ESM.pptx]

## Slide 1
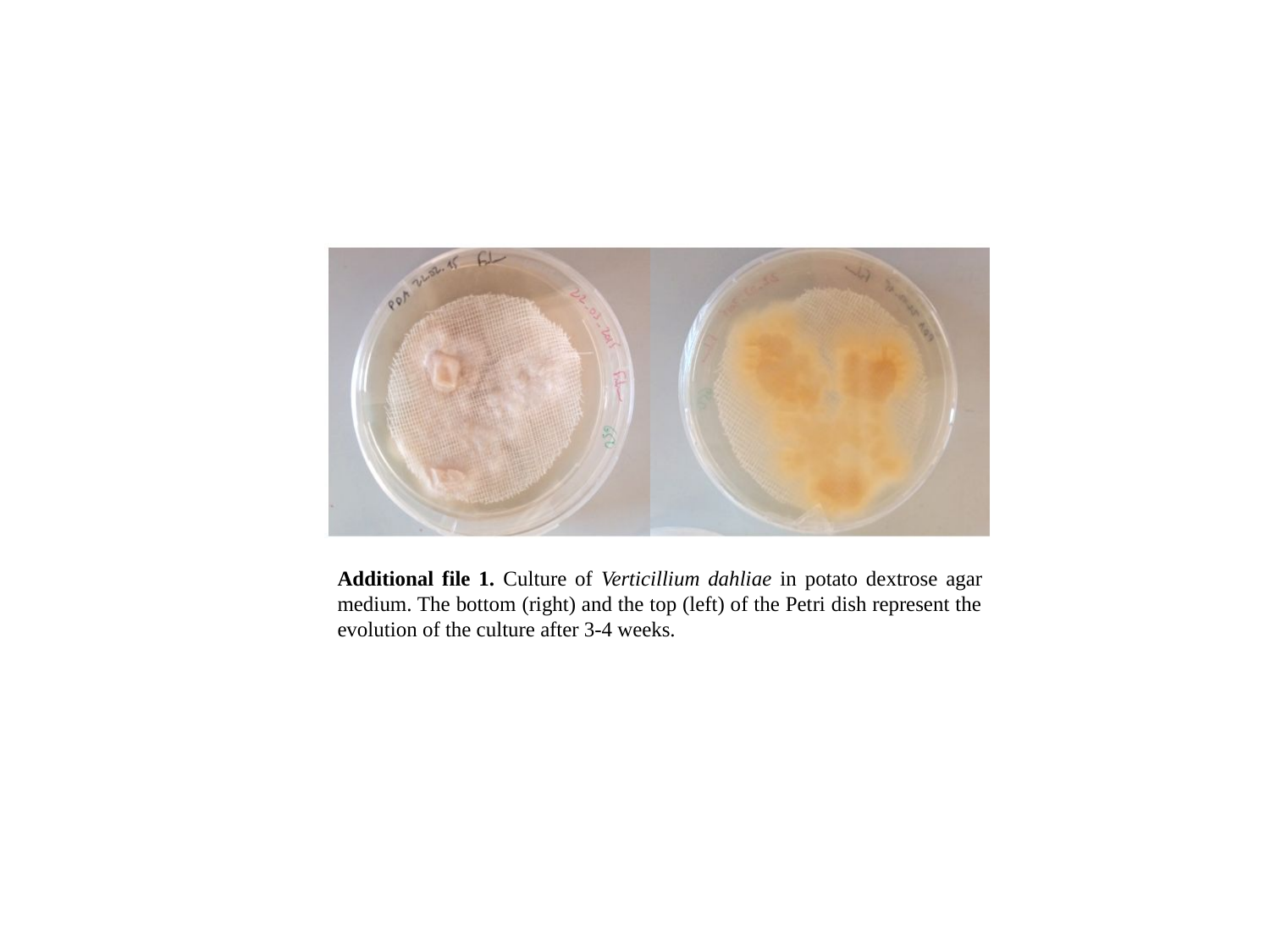

Additional file 1. Culture of Verticillium dahliae in potato dextrose agar medium. The bottom (right) and the top (left) of the Petri dish represent the evolution of the culture after 3-4 weeks.
