# Supplementary material for: Induction of PR-10 genes and metabolites in strawberry plants in response to Verticillium dahliae infection
Source: BMC Plant Biol. 2019 Apr 5;19:128. doi: 10.1186/s12870-019-1718-x (PMC6451215; doi:10.1186/s12870-019-1718-x)
Supplement: Supplementary file 2 — PR-10 nomenclature and gene number (PPTX 34 kb) [file 12870_2019_1718_MOESM2_ESM.pptx]

## Slide 1
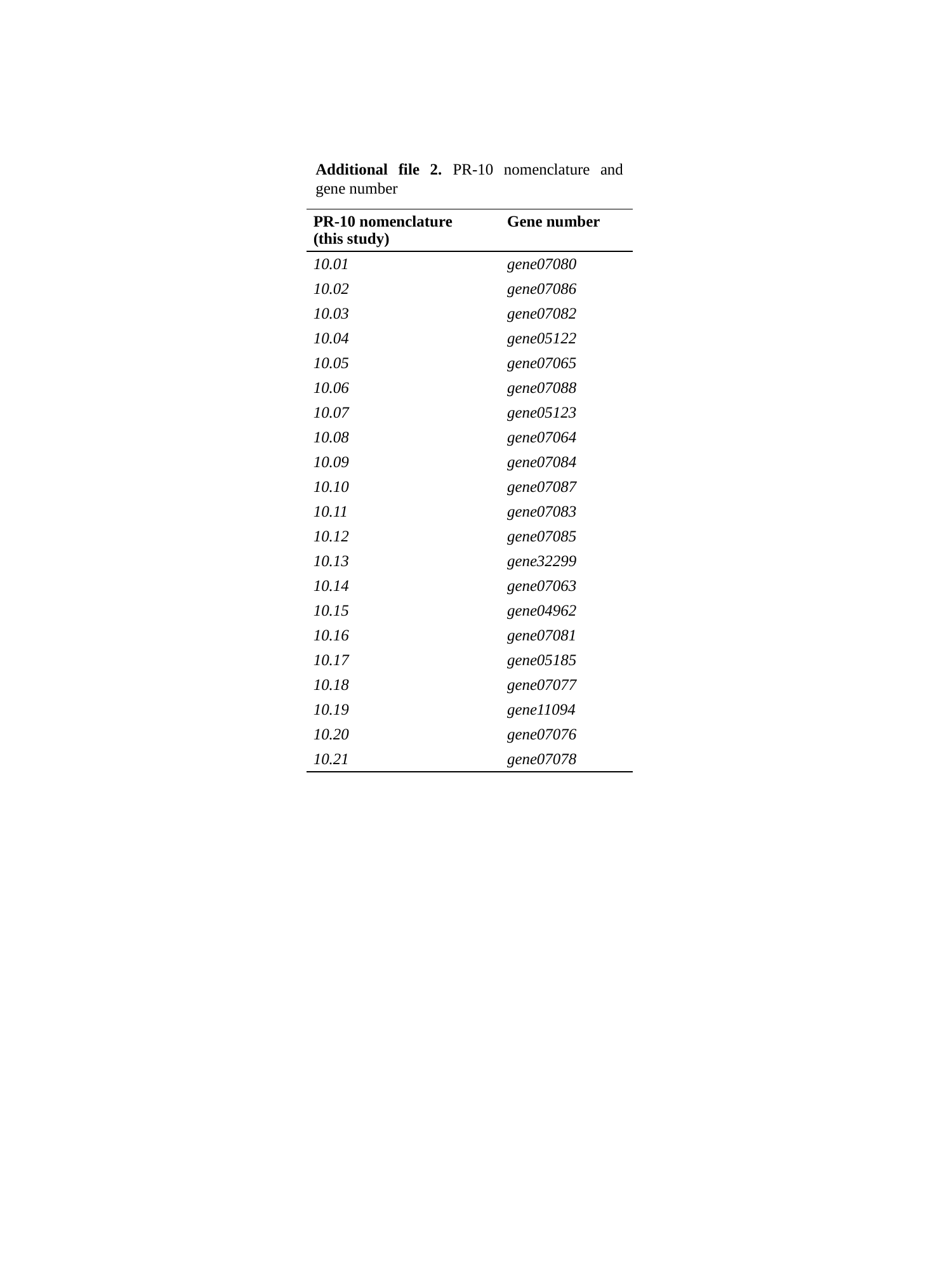

Additional file 2. PR-10 nomenclature and gene number
| PR-10 nomenclature (this study) | Gene number |
| --- | --- |
| 10.01 | gene07080 |
| 10.02 | gene07086 |
| 10.03 | gene07082 |
| 10.04 | gene05122 |
| 10.05 | gene07065 |
| 10.06 | gene07088 |
| 10.07 | gene05123 |
| 10.08 | gene07064 |
| 10.09 | gene07084 |
| 10.10 | gene07087 |
| 10.11 | gene07083 |
| 10.12 | gene07085 |
| 10.13 | gene32299 |
| 10.14 | gene07063 |
| 10.15 | gene04962 |
| 10.16 | gene07081 |
| 10.17 | gene05185 |
| 10.18 | gene07077 |
| 10.19 | gene11094 |
| 10.20 | gene07076 |
| 10.21 | gene07078 |
