# Supplementary material for: Induction of PR-10 genes and metabolites in strawberry plants in response to Verticillium dahliae infection
Source: BMC Plant Biol. 2019 Apr 5;19:128. doi: 10.1186/s12870-019-1718-x (PMC6451215; doi:10.1186/s12870-019-1718-x)
Supplement: Supplementary file 3 — Primers of the 21 PR-10 isoforms used for qPCR analysis. (PPTX 44 kb) [file 12870_2019_1718_MOESM3_ESM.pptx]

## Slide 1
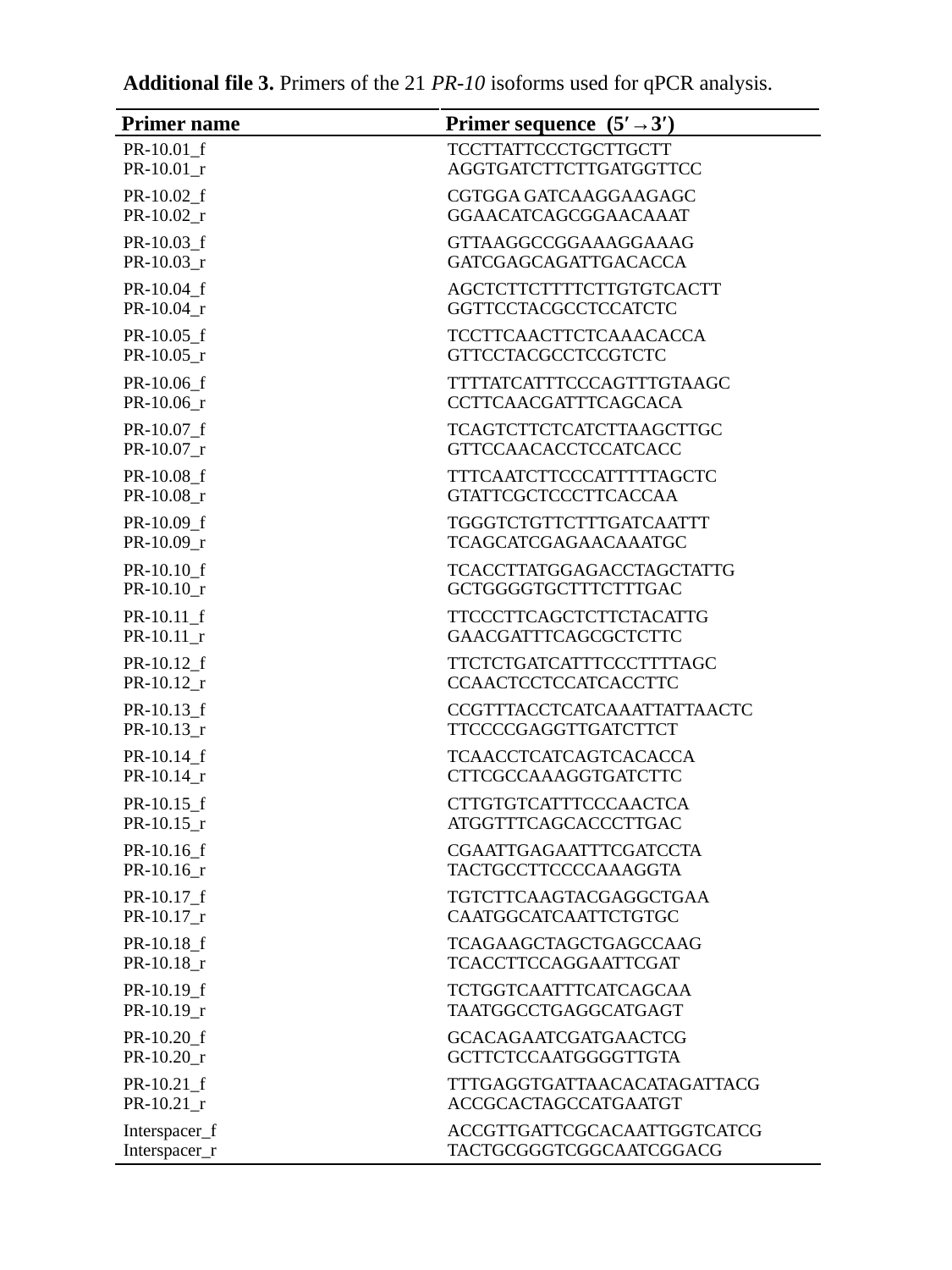

Additional file 3. Primers of the 21 PR-10 isoforms used for qPCR analysis.
| Primer name | Primer sequence  (5′→3′) |
| --- | --- |
| PR-10.01\_f PR-10.01\_r | TCCTTATTCCCTGCTTGCTT AGGTGATCTTCTTGATGGTTCC |
| PR-10.02\_f PR-10.02\_r | CGTGGA GATCAAGGAAGAGC GGAACATCAGCGGAACAAAT |
| PR-10.03\_f PR-10.03\_r | GTTAAGGCCGGAAAGGAAAG GATCGAGCAGATTGACACCA |
| PR-10.04\_f PR-10.04\_r | AGCTCTTCTTTTCTTGTGTCACTT GGTTCCTACGCCTCCATCTC |
| PR-10.05\_f PR-10.05\_r | TCCTTCAACTTCTCAAACACCA GTTCCTACGCCTCCGTCTC |
| PR-10.06\_f PR-10.06\_r | TTTTATCATTTCCCAGTTTGTAAGC CCTTCAACGATTTCAGCACA |
| PR-10.07\_f PR-10.07\_r | TCAGTCTTCTCATCTTAAGCTTGC GTTCCAACACCTCCATCACC |
| PR-10.08\_f PR-10.08\_r | TTTCAATCTTCCCATTTTTAGCTC GTATTCGCTCCCTTCACCAA |
| PR-10.09\_f PR-10.09\_r | TGGGTCTGTTCTTTGATCAATTT TCAGCATCGAGAACAAATGC |
| PR-10.10\_f PR-10.10\_r | TCACCTTATGGAGACCTAGCTATTG GCTGGGGTGCTTTCTTTGAC |
| PR-10.11\_f PR-10.11\_r | TTCCCTTCAGCTCTTCTACATTG GAACGATTTCAGCGCTCTTC |
| PR-10.12\_f PR-10.12\_r | TTCTCTGATCATTTCCCTTTTAGC CCAACTCCTCCATCACCTTC |
| PR-10.13\_f PR-10.13\_r | CCGTTTACCTCATCAAATTATTAACTC TTCCCCGAGGTTGATCTTCT |
| PR-10.14\_f PR-10.14\_r | TCAACCTCATCAGTCACACCA CTTCGCCAAAGGTGATCTTC |
| PR-10.15\_f PR-10.15\_r | CTTGTGTCATTTCCCAACTCA ATGGTTTCAGCACCCTTGAC |
| PR-10.16\_f PR-10.16\_r | CGAATTGAGAATTTCGATCCTA TACTGCCTTCCCCAAAGGTA |
| PR-10.17\_f PR-10.17\_r | TGTCTTCAAGTACGAGGCTGAA CAATGGCATCAATTCTGTGC |
| PR-10.18\_f PR-10.18\_r | TCAGAAGCTAGCTGAGCCAAG TCACCTTCCAGGAATTCGAT |
| PR-10.19\_f PR-10.19\_r | TCTGGTCAATTTCATCAGCAA TAATGGCCTGAGGCATGAGT |
| PR-10.20\_f PR-10.20\_r | GCACAGAATCGATGAACTCG GCTTCTCCAATGGGGTTGTA |
| PR-10.21\_f PR-10.21\_r | TTTGAGGTGATTAACACATAGATTACG ACCGCACTAGCCATGAATGT |
| Interspacer\_f Interspacer\_r | ACCGTTGATTCGCACAATTGGTCATCG TACTGCGGGTCGGCAATCGGACG |
