# Supplementary material for: Induction of PR-10 genes and metabolites in strawberry plants in response to Verticillium dahliae infection
Source: BMC Plant Biol. 2019 Apr 5;19:128. doi: 10.1186/s12870-019-1718-x (PMC6451215; doi:10.1186/s12870-019-1718-x)
Supplement: Supplementary file 4 — Phylogenetic tree of PR-10 genes and summary of the expression profile of the 21 PR-10 isoforms in untreated leaves, stems and roots of in vitro cultivated F. × ananassa plants (green bars) and infected tissues with V. dahliae (red bars) at various time points post (days) pathogen inoculation. qPCR using specific primers for PR-10 genes and the reference gene was used for the expression analysis. (PPTX 238 kb) [file 12870_2019_1718_MOESM4_ESM.pptx]

## Slide 1
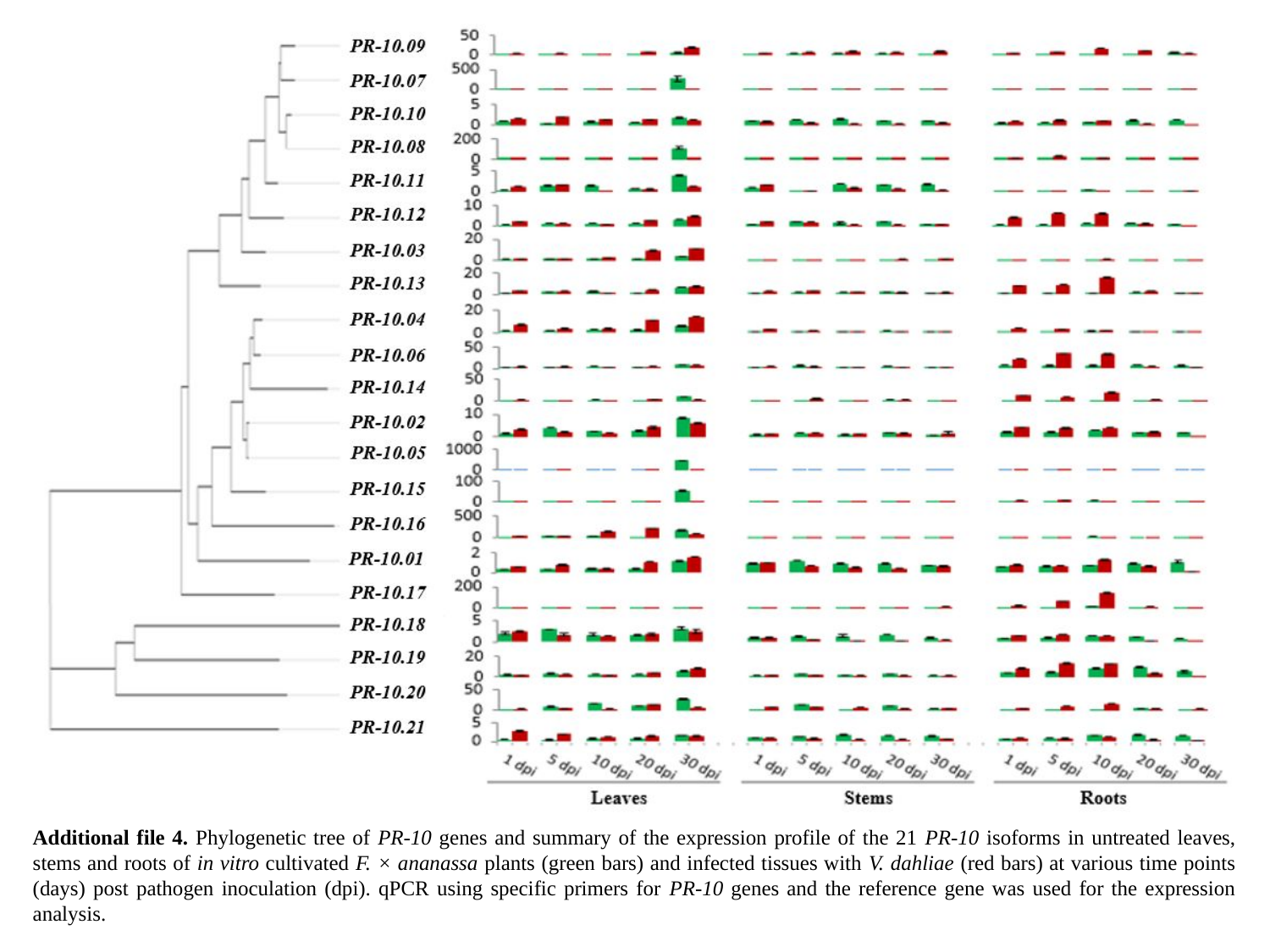

Additional file 4. Phylogenetic tree of PR-10 genes and summary of the expression profile of the 21 PR-10 isoforms in untreated leaves, stems and roots of in vitro cultivated F. × ananassa plants (green bars) and infected tissues with V. dahliae (red bars) at various time points (days) post pathogen inoculation (dpi). qPCR using specific primers for PR-10 genes and the reference gene was used for the expression analysis.
